# Robustness, dissipations and coherence of the oscillation of circadian clock: potential landscape and flux perspectives

**DOI:** 10.1186/1757-5036-1-7

**Published:** 2008-12-30

**Authors:** Jin Wang, Li Xu, Erkang Wang

**Affiliations:** 1 State Key Laboratory of Electroanalytical Chemistry, Changchun Institute of Applied Chemistry, Chinese Academy of Sciences, Changchun, Jilin, 130022, PR China; 2 Department of Physics and Astronomy, State University of New York at Stony Brook, Stony Brook, NY 11790, USA; 3 Graduate School of the Chinese Academy of Sciences, Beijing, 100039, PR China

## Abstract

Finding the global probabilistic nature of a non-equilibrium circadian clock is essential for addressing important issues of robustness and function. We have uncovered the underlying potential energy landscape of a simple cyanobacteria biochemical network, and the corresponding flux which is the driving force for the oscillation. We found that the underlying potential landscape for the oscillation in the presence of small statistical fluctuations is like an explicit ring valley or doughnut shape in the three dimensional protein concentration space. We found that the barrier height separating the oscillation ring and other area is a quantitative measure of the oscillation robustness and decreases when the fluctuations increase. We also found that the entropy production rate characterizing the dissipation or heat loss decreases as the fluctuations decrease. In addition, we found that, as the fluctuations increase, the period and the amplitude of the oscillations is more dispersed, and the phase coherence decreases. We also found that the properties from exploring the effects of the inherent chemical rate parameters on the robustness. Our approach is quite general and can be applied to other oscillatory cellular network.

**PACS Codes:** 87.18.-h, 87.18.Vf, 87.18.Yt

## 1.Introduction

Circadian rhythms are an intracellular timing mechanism, widespread in living organisms, with a period of about 24 h, which fits the day/night alterations of the Earth adapting to the fluctuating environment. In Neurospora, Arabidopsos, Drosophila, and mammals, transcription-translation-derived oscillations originating from negative feed back regulation of clock genes have been modeled at the molecular level. The study of the oscillation behavior in an integrated and coherent way is crucial in modern systems biology for understanding how these rhythms function biologically. The underlying natures of the rhythmic behavior have been explored by many experimental and theoretical methods[1,2]. However, there are so far limited theoretical studies to explain biological oscillation behavior from global and physical perspectives.

**Figure 1:**
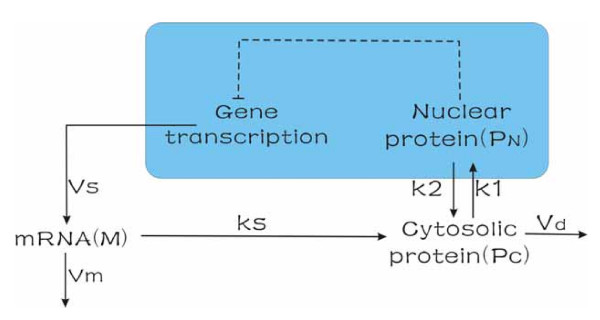
**Model**. Model for the molecular mechanism of circadian rhythms in Drosophila.

We decided to explore an established basic model based on known biological and biochemical features of a circadian clock which has negative regulation [1,2]. In this system, the PER protein represses the transcription of its own gene, per. The core model for this circadian rhythm is shown in Fig. 1. The per gene is expressed in the nucleus and transcribed into per messenger (*mRNA*). Next, the *mRNA *is transported into the cytosol and translated into PER protein (*Pc*). The protein is then transported into the nucleus and becomes the nuclear form *P*_*N *_in a reversible manner. Finally, *P*_*N *_represses the transcription of the gene. Effectively, therefore, the network is like a self repression with time delay, in which oscillation behavior is expected.

It is important to demonstrate the robustness and stability issues of the circadian system and associated oscillation patterns. There are many possible states in the systems, and it is difficult to explore all of them and the associated global nature of the network [3-12]. Fortunately, not all the states have the same weights or probabilities of occurring, due to the intrinsic statistical fluctuations from the finite number of molecules in the cell and external fluctuations from highly dynamical and inhomogeneous environments in the cell [13-20].

Therefore, instead of the averaged deterministic network of chemical rate equations, we developed a probabilistic description to model the corresponding cellular process taking into accounts of the intrinsic and external fluctuations. This can be realized by constructing a master equation for the intrinsic fluctuations or the diffusion equation for external fluctuations of the time dependent evolution probability rather than the average concentration for the corresponding deterministic chemical reaction network equations[16,21-25]. Even for the intrinsic fluctuations, we can simplify the master equation into a Fokker-Plank diffusion-like equation in the weak noise limit representing typical kinetic Markovian behavior with concentration dependent diffusion coefficients[26]. So here we use the diffusion equation to approximate the system probabilistically under the influence of either internal or external fluctuations.

By solving the Fokker-Plank diffusion equation, we can obtain the probability distribution in protein concentrations evolving in time. We can also uncover the long-time steady-state probability of this chemical reaction network in analogy to the equilibrium system, where the global thermodynamic properties can be explored using the inherent interaction potentials. We will study the global stability by exploring the underlying potential landscape for the above-mentioned non-equilibrium protein network. The generalized potential energy can be shown to be closely associated with the steady state probability of the non-equilibrium network in general and has been applied to a few systems[3-12,21,22,27-29]. Once the network problem is formulated in terms of the generalized potential function or potential landscape, the issue of the global stability or robustness is much easier to address[8,30]. We notice that although the individual averaged deterministic trajectories of a non-linear chemical reaction system might be very chaotic and complex, the corresponding statistical probabilistic distributions or the underlying landscapes which are dictated by the linear evolution equations (master equations or diffusion equations), are usually quite ordered and can often be characterized globally.

The adaptive landscape idea was first introduced into biology by S. Wright, Delbruck and Waddington [31-34]. However, the link between the dynamics and the probabilistic landscape is not clear in that work. Energy landscape ideas were pushed forward by Hans Frauenfelder [35] on protein dynamics and then P. G. Wolynes and J. N. Onuchic [36] on protein folding and interactions [37]. These ideas on proteins were based on an equilibrium approach and on knowing the potentials a priori. For a non-equilibrium system, the potential landscape is not known a priori and needs to be uncovered. In fact it is the purpose of this paper to study the global robustness of oscillation with respect to the fluctuations in the cell, directly using the properties of the non-equilibrium potential landscape, which is linked to the steady state probability of the network. This provides a basis for exploring the global and physical mechanism of biochemical oscillation.

A deterministic mathematical model of this protein clock constrained by experimental data has been proposed recently [2]. For the protein network, based on Michaelis-Menten enzyme kinetic equations, one can derive a set of differential equations which describe the variation rate of each component's concentration in the network. This leads to three independent simplified equations [2]:

(1) dMdt=vskInkIn+PNn−vmMkm+M=F1(M,Pc,PN)

(2) dPcdt=ksM−vdPckd+Pc−k1Pc+k2PN=F2(M,Pc,PN)

(3) $$\frac{dP_{N}}{dt}=k_{1}Pc-k_{2}P_{N}=F_{3}(M,Pc,P_{N})$$

where *M *is the concentration of of the clock gene *mRNA*, *Pc *is the concentration of the cytosolic protein, and *P*_*N *_is the concentration of the nuclear forms of the clock protein. The parameter *v*_*s *_represents maximum rate of transcription, and *v*_*m *_is the maximum rate of transfer into the cytosol, with the Michaelis constant *k*_*m*_. *kI *is the threshold beyond which the nuclear protein represses the transcription of per gene. The Hill coefficient *n *characterizes this repression. *k*_*s *_is the rate of protein synthesis, and *v*_*d *_is the maximum rate of protein degradation, with Michaelis constant *k*_*d*_. *k*_1 _and *k*_2 _are the first-order rate characterizing the transport of the protein into and out of the nucleus. The negative autoregulatory feedback is the origin of the oscillations.

As mentioned, the statistical fluctuations may be significant from both internal and external sources [13-20] and in general can not be ignored. We can now formulate the Fokker-Plank diffusion equation for the time evolution of the probability distributions of protein concentrations for *M, Pc *and *P*_*N*_:

(4) $$\begin{array}{lll} \frac{\partial P(M,Pc,P_{N},t)}{\partial t} & = & -\frac{\partial }{\partial M}[F_{1}(M,Pc,P_{N})*P]-\frac{\partial }{\partial Pc}[F_{2}(M,Pc,P_{N})*P]\\ & & -\frac{\partial }{\partial P_{N}}[F_{3}(M,Pc,P_{N})*P]+D*(\frac{\partial^{2}P}{\partial M^{2}})\\ & & +D*(\frac{\partial^{2}P}{\partial Pc^{2}})+D*(\frac{\partial^{2}P}{\partial P_{N}^{2}}) \end{array}$$

where *D *is the diffusion coefficient tensor(or matrix); here we use a uniform isotropic diagonal matrix for simplicity. We set vector **x **= (*M, Pc, P*_*N*_). We can rewrite the diffusion equation as $\frac{\partial P}{\partial t}$ + ▽·*J*(**x**, *t*) = 0 and define the flux vector of the system as:

(5) $$J(x,t)=FP-D\frac{\partial }{\partial x}P$$

or in the component notation, *J*_1_(*M*, *Pc*, *P*_*N*_, *t*) = *F*_1_(*M*, *Pc*, *P*_*N*_)*P *- $D\frac{\partial }{\partial M}P$, *J*_2_(*M*, *Pc*, *P*_*N*_, *t*) = *F*_2_(*M*, *Pc*, *P*_*N*_)*P *- $D\frac{\partial }{\partial Pc}P$ and *J*_3_(*M*, *Pc*, *P*_*N*_, *t*) = *F*_3_(*M*, *Pc*, *P*_*N*_)*P *- $D\frac{\partial }{\partial P_{N}}P\dot{\text{J}}$ is the steady state probability flux when ∇·*J*(**x**, *t*) = 0. It is obvious that in the steady state the divergence of *J *must vanish. One can not conclude, however, that *J *itself must vanish. Only in the equilibrium situation where the systems satisfy detailed balance, *J *= 0. For the non-equilibrium system in general, the steady state contains a circulating flow with nonzero curl. This is because $J_{ss}=FP_{ss}-D\cdot \frac{\partial }{\partial x}P_{ss}$. Therefore, $F=D\cdot \frac{\partial }{\partial x}P_{ss}/P_{ss}+J_{ss}/P_{ss}=-D\cdot \frac{\partial }{\partial x}(-\ln P_{ss})+J_{ss}/P_{ss}=-D\cdot \frac{\partial }{\partial x}U+J_{ss}/P_{ss})$. *P*_*ss *_stands for steady state probability. Although **F **in general can not be represented as a potential gradient, the driving force for the dynamics can be decomposed to two terms for non-equilibrium network systems: one is associated with the gradient of a potential closely linked to the steady state probability and the other is associated with a divergent free field. The divergent free field has no sources or sinks to start or end the force lines and therefore is recurrent or rotational in nature [12].

Once we solve for the steady state probability from the probabilistic diffusion equation, we can study the underlying properties of the potential (or potential landscape) by the relation: *U*(**x**) = -ln *P*(**x**) [4,6-12,22,28]. We use this relationship for our non-equilibrium systems (with no detailed balance, or equivalently *F *≠ -∇*U*) in analogy with equilibrium systems. However, unlike in equilibrium systems where only the steady-state probability is needed to characterize the global properties of the whole system, in non-equilibrium systems both the underlying landscape and the associated flux are essential for characterizing the global steady state properties as well as the dynamics of the protein network.

## 2. Results and discussion

The parameter values are *v*_*s *_= 0.5 *nMh*^-1^, *v*_*m *_= 0.3 *nMh*^-1^, *v*_*d *_= 1.5 *nMh*^-1^, *k*_*s *_= 2.0 *h*^-1^, *k*_1 _= 0.2 *h*^-1^, *k*_2 _= 0.2 *h*^-1^, *k*_*m *_= 0.2 *nM, kI *= 2.0 *nM, k*_*d *_= 0.1 *nM*, *n *= 4. The limit cycle for these values is attractive.

We solve the Fokker-plank equation using both the reflecting boundary condition J = 0 and the absorbing boundary condition. The results are similar; we choose the reflecting boundary condition in this paper. With certain initial conditions(both homogeneous and inhomogeneous), we obtain the steady probability distribution solution *P*_*ss *_using the finite difference method at the long time limit. Then, we can use *U*(**x**) = -ln *P*_*ss*_(**x**) to get the generalized non-equilibrium potential function (landscape) of the circadian clock.

In order to see the results clearly, we can integrate the three dimensional probability *P*(*M, Pc, P*_*N*_, *t *→ *∞*) to reduce the dimension to two. We use the formulas:

(6) $$\begin{array}{c} P_{M}(Pc,P_{N},t\to \infty )=\int_{M}P(M,Pc,P_{N},t\to \infty )dM\\ P_{Pc}(M,P_{N},t\to \infty )=\int_{Pc}P(M,Pc,P_{N},t\to \infty )dPc\\ P_{P_{N}}(M,Pc,t\to \infty )=\int_{P_{N}}P(M,Pc,P_{N},t\to \infty )dP_{N} \end{array}$$

**Figure 2:**
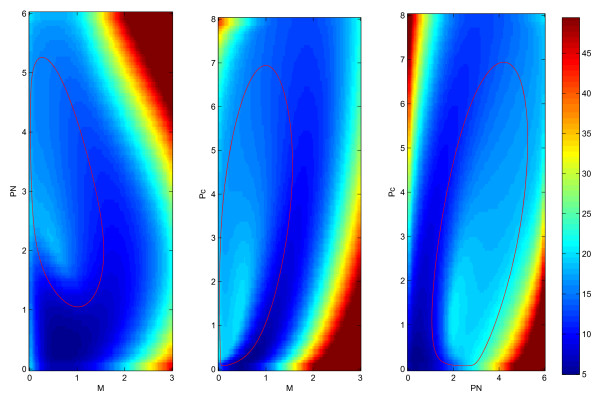
**Integrated 2 dimensional potential landscape**. The integrated two dimensional effective landscapes for the three dimensional system.

The integrated results are shown in Fig. 2. The red solid lines represent the deterministic solution of the system. We can see the potential landscape is an irregular inhomogeneous ring (the values of the potentials are represented in different colors with lower potentials in blue color) or Mexican hat like shape along the determined trajectory.

**Figure 3:**
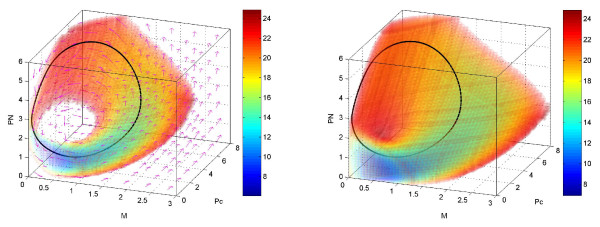
**3 dimensional potential landscape**. The three dimensional potential landscape and flux for *D *= 1*e *- 5 (A) on the left panel and potential landscape for *D *= 1*e *- 3 (B) on the right panel.

Fig. 3A (left panel) shows that the potential landscape U has a distinct closed irregular and inhomogeneous closed doughnut-like shape. In order to see clearly, we only plot only where *U *≤ 25, while *U *> 25 is transparent. The closed doughnut is around the deterministic solution which represents the lower potential and corresponding higher probability along the oscillation trajectories. The potential is higher (and the probability is lower) outside the doughnut; this means the system is attracted to the doughnut. We found that the potential landscape distributes along the oscillation ring inhomogeneously. The potential is lower for states at which the system stays longer, which is determined by the speed at which the system passes through each state in the averaged deterministic oscillation. So the potential landscape and the steady state probability along the oscillation are not uniform due to the inhomogeneity of the time spent on each state (or the passing speed at that state) of the oscillation ring. As shown in Fig. 3B (right panel), we also observe the doughnut of the potential landscape is thicker or fatter and the values of the potential landscape along the limit cycle become comparable to or even smaller than the outside of the limit cycle when the diffusion coefficient *D *increases. A further increase in the fluctuations will eventually destroy circadian rhythmicity. This is because the attraction of the limit cycle becomes weaker and the time spent on the limit cycle becomes shorter. The system transforms from a clear, robust oscillation under small fluctuations to no oscillation under high fluctuations.

**Figure 4:**
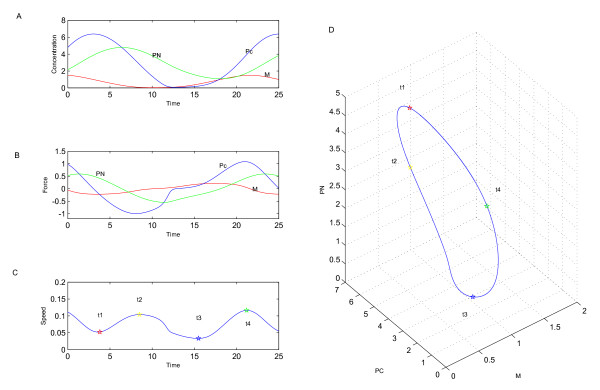
**Speed**. A: the deterministic oscillation for the three variables *M, Pc *and *P*_*N *_over a period. B: the forces of *M, Pc *and *P*_*N *_over the period. C: the speed along the cycle with time. The 'star' time parameters are as follows:*t*_1 _= 3.8, *t*_2 _= 8.5, *t*_3 _= 15.5, *t*_4 _= 21.2. D:The speed along a limit cycle: the 'star' time parameters are the same as Fig. 4C.

We can clearly see the probability distribution is not distributed evenly along the limit cycle. In order to know the nature of attractive nature of the limit cycle, we have to observe the dynamics of the network. The deterministic oscillation for the three variables *M*, *Pc*, and *P*_*N *_over a period are shown in Fig. 4(A). The forces on *M*, *Pc*, and *P*_*N *_over the period are shown in Fig. 4(B). The speed along the cycle is shown in Fig. 4(C). Fig. 4(D) shows the corresponding limit cycle with the time marks. The sign 'star' on the limit cycle shows where the values of the force and the speed have been denoted at given times. The speed along the limit cycle has two maxima, at which the amount of time spent will be smaller than at other part of the phase space. Thus, the steady probability distribution is larger at the slower speed[1].

The divergence of the flux is equal to zero at steady state. In an equilibrium system, the flux **J **= 0 (detailed balance). But in a non-equilibrium system, the flux is a curl field (*J *= ∇ × *A *in three dimensions where A is a vector field). Fig. 3(A) (left panel) shows the probability flux on the closed ring landscape of the limit cycle. We can see clearly the direction of the flux near the ring is parallel to the oscillation path, like a curl.

So the flux force is the driving force for the oscillation. The potential landscape attracts the system to the closed ring and the flux force keeps the probability flow along the ring, providing the driving force for oscillation. We can see that the flux force plays a more important role along the closed ring than outside the ring because of large ∇*U*-type forces. Therefore, the interplay of the landscape and the flux force is the most important characteristic for a non-equilibrium system.

**Figure 5:**
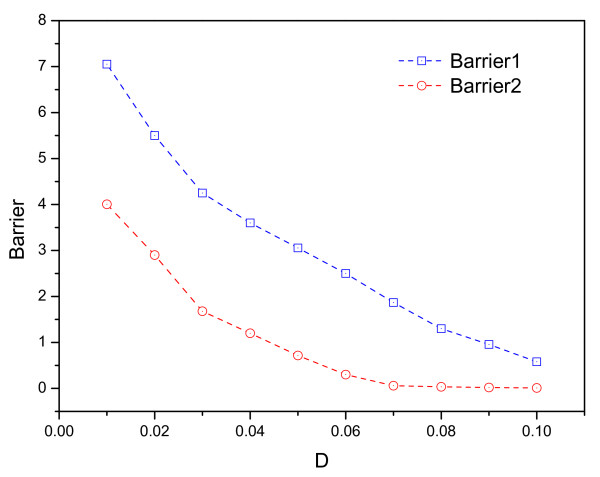
**Barrier**. The barrier height *Barrier*1 = *U*_*fix *_- *U*_*min *_and *Barrier*2 = *U*_*fix *_- *U*_*max *_versus diffusion coefficient *D*.

We can explore the global stability and robustness of the circadian clock when we obtain the potential landscape. The barrier height represents the system escaping from the oscillation attractor. Fig. 5 shows the barrier height versus the diffusion coefficient *D*. *Barrier*1 is equivalent to *U*_*fix *_minus *U*_*max*_, and *Barrier*2 is equivalent to *U*_*fix *_minus *U*_*min*_, where *U*_*fix *_is the potential local maximum inside the limit cycle; *U*_*max *_is the potential maximum along the limit cycle; and *U*_*min *_is the potential minimum along the limit cycle. We can see the barrier height becomes larger when the fluctuations decrease. It is harder for the system to go from the doughnut of attraction to outside when fluctuations are small. This means the doughnut shape of the landscape is robust, and a stable oscillation is essentially guaranteed for small fluctuations. It also implies that the barrier height can be used as a quantitative measure of the stability and robustness of the network oscillations.

In a non-equilibrium open system, there are constant exchanges of energy and information with the outside environment. This results in the dissipation of energy, which gives a global physical characterization of the non-equilibrium system. The circadian clock is a non-equilibrium open system. In the non-equilibrium steady state, the system still dissipates energy and entropy which can be determined using the landscape and the flux globally, where the entropy production rate is equal to heat dissipation. In the steady state, the dissipation of energy is closely associated with the entropy production rate. The entropy formula for the system is given as [38]

(7) *S *= -*k*_*B *_∫ *P*(**x**, *t*)ln *P*(**x**, *t*)*dx*.

**Figure 6:**
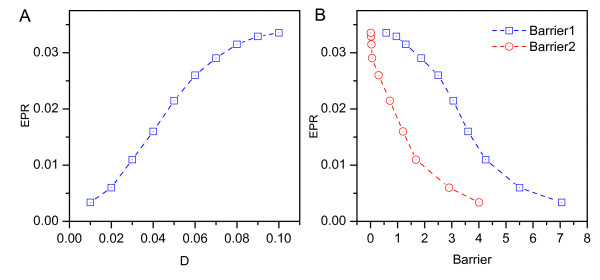
**EPR**. A:The diffusion coefficient *D *versus the entropy production rate. B:The barrier height *Barrier*1 = *U*_*fix *_- *U*_*min *_and *Barrier*2 = *U*_*fix *_- *U*_*max *_versus the entropy production rate.

By differentiating the above function, the increase of the entropy at constant temperature *T *is shown as follows:

(8) $$\begin{array}{c} T\dot{S}=k_{B}*T\int (\ln P+1)\nabla \cdot Jdx\\ =-\int (k_{B}T\nabla \ln P-F)\cdot Jdx-\int F\cdot Jdx \end{array}$$

where *e*_*p *_= -∫(*k*_*B*_*T*∇ ln *P *- **F**)·**J ***dx *is the entropy production rate [38], and *h*_*d *_= ∫ **F**·**J ***dx *is the mean rate of the heat dissipation. For a steady state, $\dot{S}$ = 0, and the entropy production *e*_*p *_is equal to the heat dissipation *h*_*d*_. In Fig. 6(A), we can see the dissipation (entropy production rate) decrease as the diffusion coefficient characterizing the fluctuations decreases; this shows that robust oscillation with less fluctuation dissipates less energy and is more stable. From Fig. 6(B), we also find that less dissipation leads to higher barrier heights for escaping from the oscillation cycle and therefore a more stable network. So, minimization of the dissipation cost might serve as a design principle for evolution of the network because the entropy production is a global characterization of the circadian network; it is intimately related to the robustness of the network.

**Figure 7:**
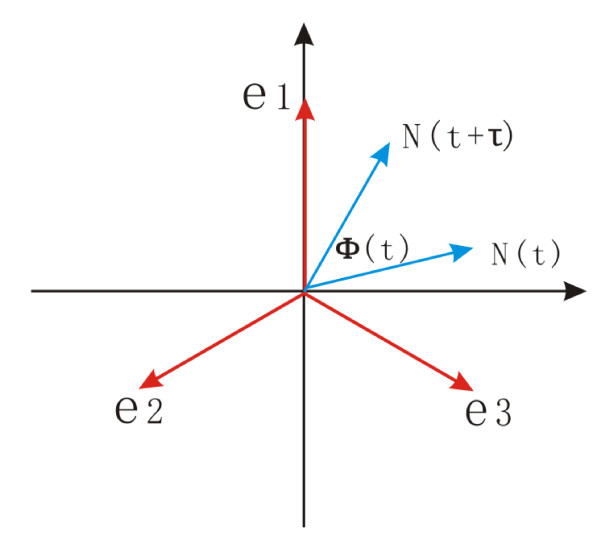
**Definition of phase coherence**.

**Figure 8:**
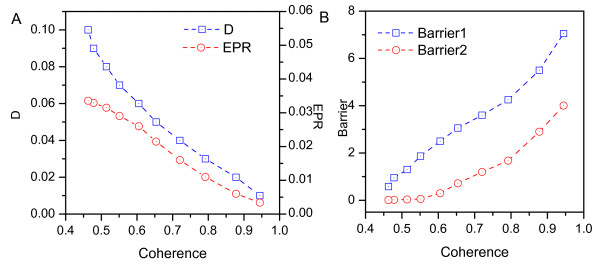
**Coherence**. A: The coherence versus the diffusion coefficient *D *and entropy production rate. B: The coherence versus the Barrier height.

The robustness of the oscillation with respect to the diffusion coefficient *D *can be quantified further by the phase coherence *ξ*, which measures the degree of periodicity of the time evolution of a given variable[39]. The phase coherence *ξ *quantitatively measures the degree of persistence of the oscillatory phase, and is defined as follows: First, the vector *N*(*t*) = *n*_1_(*t*)*e*_1 _+ *n*_2_(*t*)*e*_2 _+ *n*_3_(*t*)*e*_3 _is shown in Fig. 7. The unit vectors are *e*_1 _= (0, 1), *e*_2 _= (-$\sqrt{3}$/2, -1/2) and *e*_3 _= (-$\sqrt{3}$/2, 1/2), where *n*_1_(*t*), *n*_2_(*t*), and *n*_3_(*t*) are the concentrations of the three kinds of protein molecules at time *t*. *φ*(*t*) is the phase angle between *N*(*t*) and *N*(*t *+ *τ*), where *τ *should be smaller than the deterministic period and larger than the fast fluctuations. We choose *τ *= 0.2 *k*^-1^. The oscillation goes in the positive orientation (counterclockwise), so *φ*(*t*) > 0. The formula for *ξ *is then:

(9) $$\xi =\frac{2\sum_{i}\theta (\phi (t))\phi (t)}{\sum_{i}\left|\phi (t)\right|}-1$$

where *θ*(*φ*) = 1 when *φ*(*t*) > 0, and *θ*(*φ*) = 0 when *φ*(*t*) ≤ 0, and sums are taken over every time step for the simulated trajectory. *ξ *≈ 0 means the system moves stochastically and has no coherence. The oscillation is most coherent when *ξ *is close to 1. The value of *ξ *becomes larger when the fluctuations are smaller, since the trajectories are more periodic in their evolution. Fig. 8(A) shows *ξ *decreases as the the diffusion coefficient increases, implying that the coherence of the oscillation can be destroyed by fluctuations. Conversely, less fluctuation yields a more coherent, robust, and stable system. We also see that *ξ *becomes larger with a lower heat loss or entropy production rate, and conclude that less dissipation leads to more coherence. We further see that *ξ *increases with barrier height (Fig. 8(B)). This shows that a less dissipated network tends to preserve the coherence of the oscillations.

**Figure 9:**
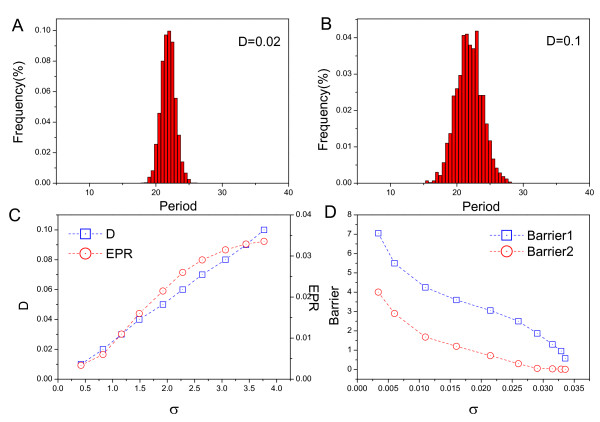
**Period**. A: The period distribution for *D *= 0.02. B: The period distribution for *D *= 0.1. C: The diffusion coefficient *D *versus the standard deviation of period *σ*_*Period *_and the entropy production rate. D: The standard deviation of period *σ*_*Period *_versus the barrier height.

We can also use stochastic simulations for various values of *D *to illustrate the robustness of circadian oscillation. We solve the chemical reaction network equations under the fluctuations which reflect external noise. To assess the effect of molecular noise on circadian oscillations, we have used stochastic Brownian dynamics to perform stochastic simulations of the deterministic model governed by equations(1–3). Fig. (9A and 9B) shows the distributions of the period of oscillations calculated for 2000 successive cycles. We can see that, when the fluctuations increase, the distribution becomes more spread out, but the mean period and mean amplitude are still close to the deterministic period of the oscillations. In Fig. 9(C), the standard deviation *σ *from the mean increases when the fluctuations increase[1,2]. This implies that less fluctuations lead to more coherent oscillations. We also see that the period distribution becomes less dispersed when the entropy production rate decreases. This shows that a less dissipated network can lead to a more coherent oscillation with a unique period instead of a distribution of periods. In Fig. 9(D), we see that higher barrier heights lead to less dispersed period distributions.

**Figure 10:**
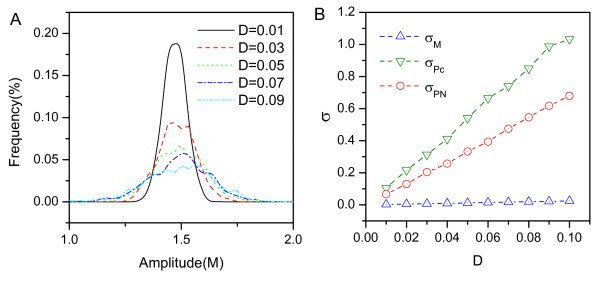
**Amplitude**. A: The amplitude distribution with different *D*. B: The standard deviation of amplitude *σ *versus the *D*.

We also show the distributions of the amplitude for *M *with increasing *D*. We can see the distribution becomes more dispersed but stays close to the deterministic value as the fluctuations increase in Fig. 10(A). The standard deviation *σ *increases when *D *goes up in Fig. 10(B), again showing less fluctuations leading to more robust oscillations.

**Figure 11:**
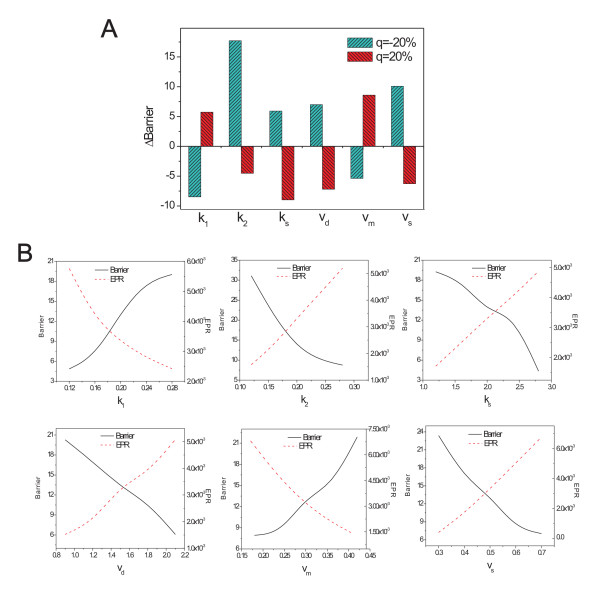
**Barrier height and entropy production versus chemical rate parameters**. A: Barrier changes with respect to changes of rate parameters (red: rate increase. green: rate decrease) *k*_1_, *k*_2_, *k*_*s*_, *v*_*m*_, *v*_*d*_, and *v*_*s*_. B. Barrier height and entropy production rate versus chemical rate parameters *k*_1_, *k*_2_, *k*_*s*_, *v*_*m*_, *v*_*d*_, and *v*_*s*_.

To explore the effects of the inherent chemical rate parameters on the robustness, we can try to find out which reactions are important, and further, which protein elements are crucial in maintaining the robustness. Fig. 11(A) shows the effects of rate parameters on the robustness. The six rate parameters increase by twenty percent (red), and decrease by twenty percent (green). The bars show the change in barrier height for different parameters. *q *is the percentage by which the rate constants are increase or decreased. Fig. 11(B) shows the barrier height (solid line) and the entropy production rate (dashed line) versus the six rate parameters. We can see that when the rate parameters *k*_1 _and *v*_*m *_increase, the barrier height increases and the entropy production rate decreases, as the system becomes more stable and robust. We can also see that when the other four rate parameters (*k*_2_, *k*_*s*_, *v*_*d*_, *v*_*s*_) increase, the barrier height decreases and the entropy production rate increases as the system becomes less stable and robust.

**Figure 12:**
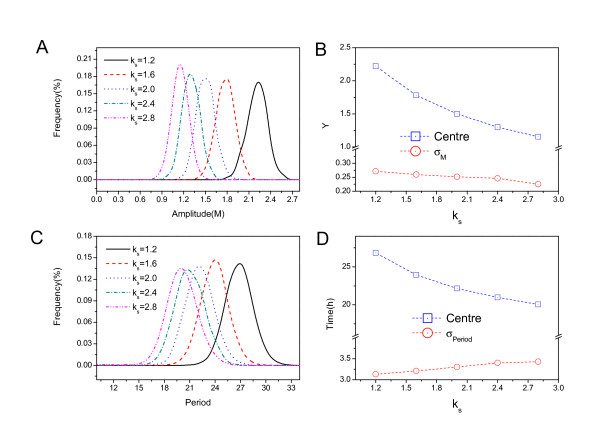
**Amplitude and period distribution changes with respect to rate *k***_*s*_. A: Amplitude distribution for different *k*_*s *_rate. B: Amplitude center and the standard deviation *σ *versus chemical rate *k*_*s*_. C: Period distribution for different *k*_*s *_rates. D: Period center and the standard deviation *σ *versus rate *k*_*s*_.

We can choose the rates *k*_*s *_and *v*_*m *_to further explore the period and amplitude using stochastic Brownian dynamics, since they represent the largest changes of the barrier height from the increasing the rate parameters. Fig. 12(A) shows the amplitude distribution for different *k*_*s *_rates. Fig. 12(B) shows the amplitude center and the standard deviation *σ *both decrease when the rate *k*_*s *_increase. Fig. 12(C) shows the period distribution for different *k*_*s *_rates and Fig. 12(D) shows the period center decrease and the standard deviation *σ *increase when the rate *k*_*s *_increase. This implies that the fluctuations in period measured by the variance increase as the *k*_*s *_increases. Therefore the network becomes less stable and coherent due to the trend of larger fluctuations.

**Figure 13:**
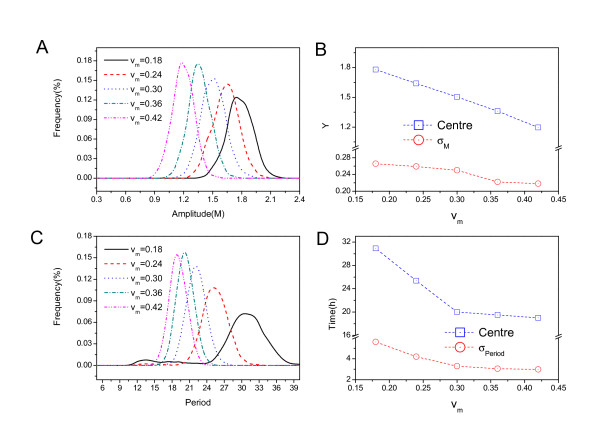
**Amplitude and period distribution changes with respect to rate *v*_*m*_**. A: Amplitude distribution for different chemical rates *v*_*m*_. B: Amplitude center and the standard deviation *σ *versus the rate *v*_*m*_. C: Period distribution for different *v*_*m *_rates. D: Period center and the standard deviation *σ *versus the rate *v*_*m*_.

Fig. 13(A) shows the amplitude distribution for different *v*_*m *_rate. Fig. 13(B) shows the amplitude center and the standard deviation *σ *both decrease when the rate *v*_*m *_increase. Fig. 13(C) shows the period distribution for different *v*_*m *_rate and Fig. 13(D) shows the period center and the standard deviation *σ *both decrease when the rate *v*_*m *_increase. This implies that the fluctuations in period and amplitude measured by the variance decrease as the *v*_*m *_increases. Therefore the network become more stable and coherent due to the trend of smaller fluctuations.

### 2.1. Mathematical material

We can study the network of chemical reactions in fluctuating environments:

(10) $$\dot{x}=F(x)+\zeta$$

where **x **= {*x*_1_(*t*), *x*_2_(*t*), ... *x*_*n*_(*t*)} is the concentration vector, with each component of which representing different protein species in the network. The **F**(**x**) = {*F*_1_(**x**), *F*_2_(**x**), ... *F*_*n*_(**x**)} is the chemical reaction rate flux vector involving the chemical reactions which are often non-linear in protein concentrations **x **(for example, enzymatic reactions). The equations $\dot{x}$ = **F**(**x**) describe the averaged dynamical evolution of the chemical reaction network (see details in the next subsection). As mentioned, in the cell, the fluctuations can be very significant from both internal and external sources [40-44] and in general can not be ignored. A term *ζ *is added mimicking these fluctuations in an assumed Gaussian distribution (from the large-number theorem in statistics). Then the auto correlations of the noise is given by:

(11) <*ζ *(**x**, *t*)*ζ*^*τ *^(**x'**, *t'*) >= 2*D*(**x**, *t*)*δ *(*t *- *t'*).

Here *δ*(*t*) is the Dirac delta function and the diffusion matrix *D *is explicitly defined by <*ζ*_*i*_(*t*)*ζ*_*j*_(*t'*) >= 2*D*_*ij*_*δ*(*t *- *t'*). The average <...> is carried out with the Gaussian distribution for the noise. Therefore, we realize that the resulting evolution trajectories of the protein concentrations are stochastic. So instead of following the determinist path of probability equal to one, we now need to quantify the probability of specific paths. The probabilistic description is more appropriate for the system under fluctuating environments. The probabilistic evolution follows a Fokker-Planck diffusion equation as discussed in the main text.

We can explore the long time steady state properties and collect the statistics to obtain the steady state distribution function *P*_0_(**x**) for the state variable **x **(representing the protein concentrations of the protein network in this case). In the equilibrium systems where a potential U where the force is a gradient of it, *P*_0_(**x**) is exponentially related to potential energy function *U*(**x**). So we obtain the information of steady state probability from U. For the non-equilibrium system, we do not know the information of the potential a priori. But we can obtain the information of the steady state probability by solving the probabilistic evolution equation and taking the long time limit. In analogy with the equilibrium system, we can define the generalized potential U for the non-equilibrium case from the steady state probability [4,6,7,9,10,22,28]:

(12) $$P_{0}(x)=\frac{1}{Z}\exp\{-U(x)\},$$

with the partition function *Z *= ∫ *d***x **exp{-*U*(**x**)}. The rational for the definition of the non-equilibrium potential this way is given earlier in the main text due to the driving force (for the dynamics) decomposition as gradient of a potential and curl flux. From the steady-state distribution function, we can therefore identify U as the generalized potential function of the network system. In this way, we map out the potential landscape. Once we have the potential landscape, we can discuss the global stability of the protein cellular networks.

## 3. Conclusion

We have shown that we can explore the global features of the circadian rhythms model. Finding the potential landscape and associated flux is the key to addressing the robustness issue of the networks. We have uncovered the underlying potential landscape of a circadian clock. This is realized by explicitly constructing the probability of the states of the protein network by solving the corresponding probabilistic diffusion equation. The landscape of the oscillation has an irregular and inhomogeneous closed ring valley or doughnut-like shape. We also found that the flux along the cycle path is the driving force for coherent oscillation. The potential barrier height for escaping from the limit cycle attractor determines the robustness and stability of the network oscillations. We found as the diffusion coefficient becomes smaller, the potential barrier becomes greater, and furthermore the statistical fluctuations are effectively more severely suppressed. This leads to robustness of the biological limit cycle basin of the protein network.

We observe the global dissipation in terms of the entropy production of the whole system increases when the diffusion coefficient *D *increases. The period and the amplitude distribution becomes widely dispersed when *D *increases, and the phase coherence decreases. These are three ways of characterizing the robustness of the oscillation in addition to the barrier height measure from the basin of the attraction. Low entropy production might serve as a design principle for robust networks.

The robustness, coherence, and dissipation of the circadian oscillations with respect to the changes with the rate parameters can be studied as well. And we found protein element k_s _is crucial in maintaining the robustness in the network.
